# Robotic-Assisted Common Bile Duct Exploration for Choledocholithiasis: A Systematic Review and Meta-Analysis of Efficacy and Safety Outcomes

**DOI:** 10.7759/cureus.93310

**Published:** 2025-09-26

**Authors:** Teck Yon Lee, Wynne Sim, Sophia Wong Ching Hwai, Syed Ali Naqvi

**Affiliations:** 1 General Surgery, NHS Fife, Kirkcaldy, GBR; 2 Ear, Nose, and Throat (ENT), County Durham & Darlington NHS Foundation Trust, Darlington, GBR; 3 Respiratory Medicine, Northern Care Alliance NHS Foundation Trust, Greater Manchester, GBR; 4 General Surgery, Victoria Hospital Kirkcaldy, NHS Fife, Kirkcaldy, GBR

**Keywords:** cbd stone, choledocholithiasis, common bile duct exploration (cbde), common bile duct stone, minimally invasive surgery, robotic-assisted surgery, robotic biliary surgery

## Abstract

Robotic-assisted common bile duct exploration (RACBDE) shows promise as a minimally invasive option, offering better outcomes over traditional approaches. This study reviews current evidence on RACBDE outcomes and, where possible, compares it to other surgical procedures. A comprehensive search was conducted across Ovid MEDLINE, EMBASE, Cochrane, and Scopus. We reviewed 346 studies published in the last 15 years, of which eight met the inclusion criteria. Eligible studies reported safety outcomes and efficacy of RACBDE for choledocholithiasis. Studies comparing RACBDE to other modalities (endoscopic retrograde cholangiopancreatography (ERCP), laparoscopy, and open surgery) were included for comparative meta-analysis. A total of 438 patients were included. Using a random effects model, single-arm analysis of eight datasets revealed an overall pooled stone clearance rate of 95% (95% CI: 0.90, 0.98; I² = 9.16%), while the overall pooled complication rate was 25% (95% CI: 0.17, 0.33; I² = 43.45%). Comparative analysis of three datasets revealed that RACBDE had a 34% lower risk of complications compared to open surgery (log risk ratio (RR): -0.42; 95% CI: -0.78 to -0.06; p < 0.05). However, open surgery had a 37-minute shorter operation time (mean difference (MD): 37.71 minutes; 95% CI: 15.28-60.13; p < 0.05). Meta-regression with the year as a covariate showed a 0.7% reduction in the complication rate per year with RABCDE; however, this was not statistically significant (coefficient: -0.00679; p = 0.37). This study suggests that RACBDE is highly effective for stone clearance with an acceptable complication rate. Compared to open surgery, RACBDE is linked to a significantly lower complication rate but longer operative times.

## Introduction and background

Gallstone disease is highly prevalent worldwide, affecting approximately 6.1% of the global population, and is projected to increase over the next few years, with the prevalence of gallstone disease doubling in the past three decades (7.4% to 13.9%) [1]. Of those patients with gallbladder stones, 10-20% develop common bile duct (CBD) stones, and up to 3-10% of patients undergoing cholecystectomy are found to have choledocholithiasis [2,3].

The optimal management of CBD stones remains controversial. Since its emergence in the 1970s, endoscopic retrograde cholangiopancreatography (ERCP) has become the most used first-line intervention, typically paired in a “two-stage” approach with laparoscopic cholecystectomy. However, this approach necessitates multiple procedures and is associated with risks, such as post-ERCP pancreatitis, perforation of bile duct, and sphincter dysfunction [4-6].

In contrast, single-stage surgical approaches, including laparoscopic common bile duct exploration (LCBDE) and, more recently, robotic-assisted CBDE (RACBDE), have gained traction. Meta-analyses comparing one- versus two-stage approaches suggest that single-stage LCBDE offers comparable stone clearance, fewer procedures, shorter hospital stays, and similar or reduced complication rates [7,8].

RACBDE builds on these advantages, offering enhanced surgeon dexterity, three-dimensional visualization, and wristed instrument articulation, which are particularly valuable in complex biliary anatomy and difficult stone extractions [9]. Over time, improvements in robotic systems such as haptic feedback, reduced dock/setup time, and refined energy instruments have increased the safety, efficiency, and reproducibility of RACBDE, making it a promising evolution of minimally invasive bile duct surgery [10].

However, the widespread adoption of robotic platforms is limited. They are more expensive than traditional laparoscopic procedures and have not consistently demonstrated better patient outcomes to justify the extra cost. On top of that, these systems are not widely available, especially in lower-income countries.

This systematic review and meta-analysis aims to evaluate the efficacy and safety profile of RACBDE and compare its outcomes to other surgical modalities. We included single-arm and comparative analyses against open surgery to analyze key outcomes, such as stone clearance, complication rates, and operative time, highlighting the evolving role of robotics in CBD stone management.

## Review

Methods

This protocol was registered in PROSPERO with the ID (CRD420251044302). This study was conducted in accordance with The Preferred Reporting Items for Systematic reviews and Meta-Analyses (PRISMA) [11].

Eligibility Criteria

Inclusion criteria: Adults aged ≥18 years with suspected or confirmed common bile duct (CBD) stone or choledocholithiasis who underwent RACBDE as a primary or secondary treatment strategy for common bile duct stone removal. All study designs that report on efficacy and/or safety outcomes of RACBDE for choledocholithiasis were considered. This includes randomized controlled trials (RCTs), non-randomized controlled trials (NRCTs), comparative or single-arm cohort and case-control studies. Case series were considered if they provided sufficient detail to answer the research question; however, this was judged carefully by two reviewers.

Exclusion criteria: We excluded the pediatric population <18 years, pregnant population, studies with insufficient data to extract the required efficacy and/or safety outcomes, and studies involving animal models or in vitro experiments. We also excluded case reports, reviews, editorials, letters, and conference abstracts.

Search Strategy

Our search strategy included four major bibliographic databases: The Cochrane Library (CLIB), Embase (via Ovid), MEDLINE (via Ovid), and Scopus. The following keywords were used: (“Robotic” OR “robot-assisted”) AND (“Bile duct exploration” OR “bile duct stone” OR “choledocholithiasis”). For completeness, we searched gray literature, including ClinicalTrials. gov [12], using the keywords “choledocholithiasis” and “robotic surgery,” which yielded two studies. However, both studies were excluded due to a lack of posted results. We conducted the search on May 7, 2025, and only included studies published in English between January 1, 2010, and May 1, 2025.

Study Selection

**Figure 1:**
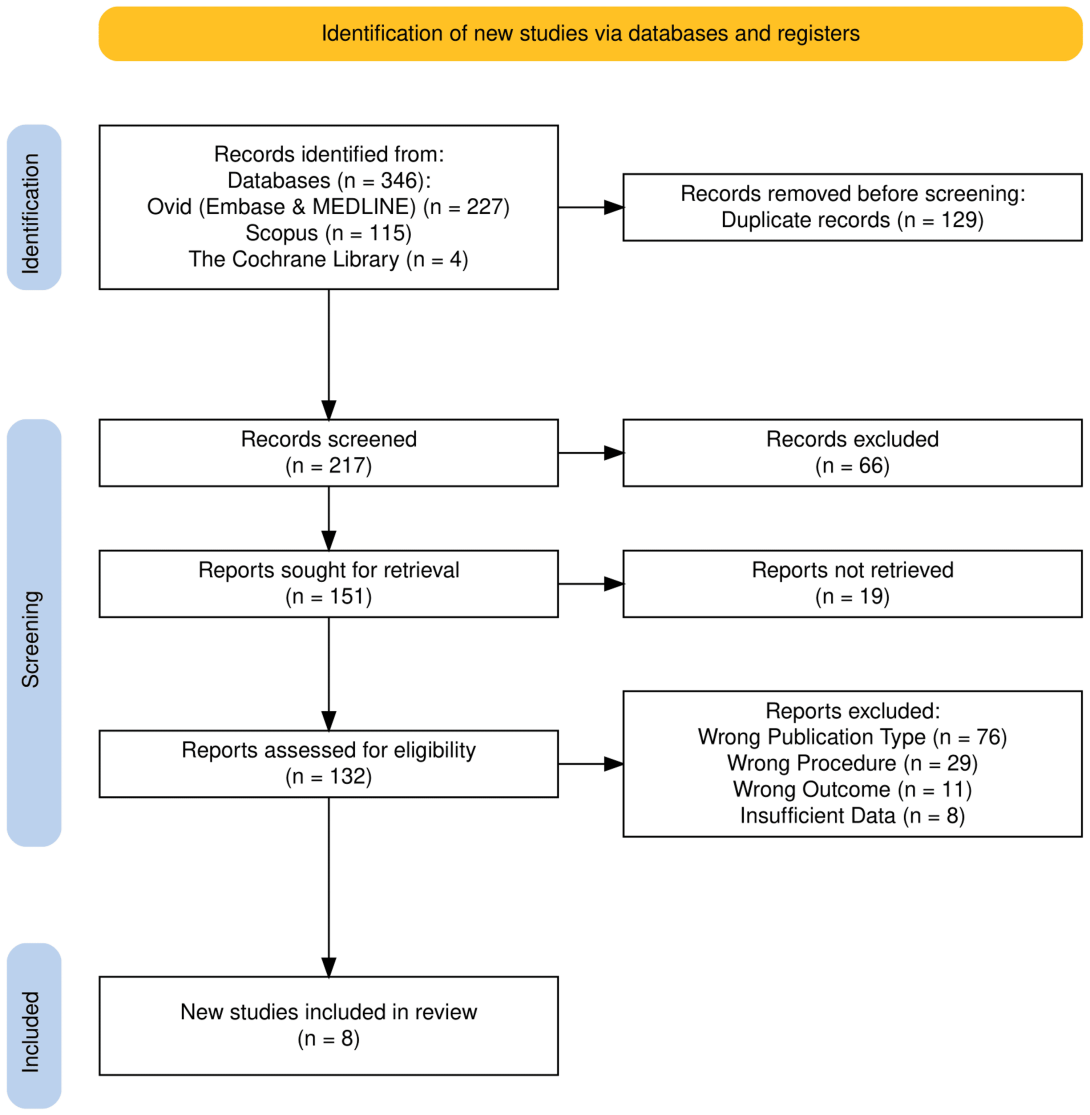
PRISMA flow diagram. PRISMA: Preferred Reporting Items for Systematic Reviews and Meta-Analyses.

Two authors (TL and WS) combined results from all databases and uploaded them to Rayyan AI® software (Rayyan Systems Inc., Cambridge, MA, USA). Duplicate records were removed prior to screening. Data screening in Rayyan AI® was blinded to each author to reduce bias. All authors independently screened titles and abstracts of identified articles for eligibility during the initial screening phase. Articles meeting the inclusion criteria were then subjected to a full-text review. Reasons for exclusion at each stage were documented and summarized in the PRISMA flow diagram (Figure 1). Any disagreements between reviewers were resolved through discussion. If consensus could not be reached, decisions were finalized by a majority vote with the third author (SW).

Data Extraction

Data extraction was performed independently by each author and subsequently verified by a second author to ensure accuracy. Extracted data were organized into two primary domains: efficacy and safety outcomes. We also collected data about study characteristics, including study ID (author and year), country, study design, surgical approach, and sample size.

Efficacy outcomes: Operative time, stone clearance rate, need for additional procedures (intraoperative conversions, readmissions, or secondary interventions), and type of surgical procedure performed.

Safety outcomes: Overall complication rate, average length of hospital stay, and specific postoperative complications, which were further classified by type (bile leak, cholangitis, pancreatitis, surgical site infection, cardiac and respiratory complications, and death). For comparator studies, the type of comparator was also collected, and data extraction followed the same domain structure. For studies that reported continuous variables as medians with interquartile ranges (IQRs), means and standard deviations (SDs) were estimated using the method proposed by Wan et al. [13], which allows for transformation of summary statistics to enable inclusion in meta-analyses.

Quality Assessment

Risk of bias was independently assessed by two reviewers (TL and WS) using the Methodological Index for Non-Randomized Studies (MINORS) tool [14]. This tool is designed for evaluating the methodological quality of non-randomized surgical studies, making it appropriate for our included studies, which mainly included non-randomized studies and lacked standardized comparators.

For single-arm studies, the first eight MINORS items were applied, yielding a maximum score of 16. For comparative studies, all 12 items were assessed, with a maximum score of 24, in accordance with the MINORS guidelines. Each item was scored as follows: “2” if adequately reported, “1” if inadequately reported, and “0” if not reported.

Based on total scores, studies were categorized into three tiers of risk: low risk of bias: ≥75% of the maximum score (≥12 for single arm; ≥18 for comparative studies); moderate risk of bias: 51-74% of the maximum score; high risk of bias: ≤50% of the maximum score.

Disagreements between reviewers were resolved through discussion or consultation with the third author. The results were visually generated using the online RobVis tool [15].

Statistical Analysis

Data analysis was performed using STATA version 16.1 (StataCorp LLC, College Station, TX) with the meta and metaprop packages. All studies were performed using a random-effects model with a 95% confidence interval (CI) for all pooled estimates.

For studies without a comparator arm, single-arm meta-analyses were conducted to estimate pooled proportions for efficacy outcomes (stone clearance rate) and safety outcomes (overall complication rate). Results were visualized using forest plots and used to make comparisons with published outcomes from other surgical modalities.

In cases where studies reported 100% event rates (100% stone clearance), the Freeman-Tukey double arcsine transformation was applied to stabilize variances and prevent distortions in pooled estimates due to zero or full proportions [16].

To explore potential sources of heterogeneity, meta-regression was performed with year of publication, sample size, operating time, and surgical approach as covariates.

For studies comparing robotic-assisted common bile duct exploration (RACBDE) to other surgical approaches, outcomes were analyzed as follows: Continuous variables (operative time) were assessed using mean differences (MD). Dichotomous variables (complication rate) were analyzed using log risk ratios (log RR), which were then back-transformed to risk ratios (RR) for interpretability.

Statistical heterogeneity among studies was assessed using the I² statistic, with values >50% considered indicative of substantial heterogeneity [17]. These values are reported in each corresponding forest plot.

Results

The initial search generated 346 studies, of which 129 duplicates were removed. An additional 209 studies were then excluded based on the criteria outlined in the PRISMA flow chart in Figure 1. This screening process resulted in the inclusion of eight studies for analysis [18-25]. These included five cohort studies, two case-control studies, and one case series with 21 patients with enough data for extraction. The studies were geographically diverse, spanning the USA, UK, Canada, China, Hong Kong, and Israel. The PRISMA flow diagram (Figure 1) summarizes the study selection process [26]. The characteristics, efficacy and safety outcomes of the included studies are summarized in Tables 1, 2. 

**Table 1:** Summary of study characteristics and demographics of included studies. RACBDE: robotic-assisted common bile duct exploration; ERCP: endoscopic retrograde cholangiopancreatography.

Study ID	Country	Study design	Surgical approach	Sample size (RACBDE)	Comparator type	Sample size (comparator arm)
Zhang et al. 2025 [25]	USA	Prospective, case series	Transcystic	21	None	
DeJesus et al. 2024 [24]	USA	Retrospective, case-control	Transcystic	53	ERCP	101
Latif et al. 2024 [23]	UK	Retrospective, cohort	Transcystic, choledochotomy	23	None	
Younis et al. 2022 [22]	Israel	Retrospective, cohort	Choledochotomy	12	Open, laparoscopic	64 (Open), 26 (laparoscopic)
Almamar et al. 2018 [21]	Canada	Retrospective, cohort	Choledochotomy	50	Open	30
Alkhamesi et al. 2012 [20]	Canada	Retrospective, case-control	Choledochotomy	19	Open	18
Chan et al. 2011 [19]	Hong Kong	Retrospective, cohort	Choledochotomy	16	None	
Ji et al. 2011 [18]	China	Prospective, cohort	Choledochotomy	5	None	

**Table 2:** Summary of efficacy and safety outcomes of included studies. SD: standard deviation; ERCP: endoscopic retrograde cholangiopancreatography; AKI: acute kidney injury.

Study ID	Procedure	Sample size	Operating time in minutes (Mean ± SD)	Stone clearance rate (number, %)	Conversions	Readmissions	Overall complication	Average length of stay (days, SD)	Type of complication
Zhang et al. 2025 [25]	Robotic	21	266 ± 143.13	19, 90.48%	0	3 (post-op ERCP)	3	Mean 5.29, SD 4.08	
DeJesus et al. 2024 [24]	Robotic	53	-	48, 91%	0	5 (post-op ERCP)	8	Mean 3.9, SD 3.2	One (anterior cutaneous nerve entrapment), one (port side hematoma with AKI), one (right hepatic duct injury), and five (retained stones)
	ERCP	101	-	90, 89 %	3: Robotic	4 (second ERCP during index admission), 32 requiring one additional ERCP after discharge	21	Mean 5.1, SD 2.3	Two (cystic stump leak), three (pancreatitis), two (surgical site infection), two (abscess), one (biliary stent occlusion), 11 (retained stones)
Latif et al. 2024 [23]	Robotic	23	174 ± 72.6	23, 100%	0	2 (one subhepatic collection requiring laparoscopy washout, one laparoscopic suture repair of hernia)	8	Mean 2, SD 2.96	One (cholangitis), one (abscess), and one (hernia)
Younis et al. 2022 [22]	Robotic	12	324 ± 69	12, 100%	1: Open	0	5	Mean 6, SD 2	
	Laparoscopic	26	289.2 ± 52	26, 100%	8: Open	2	12	Mean 7, SD 3	One (death)
	Open	64	306 ± 116	64, 100%	0	7	44	Mean 11, SD 3	21 (surgical site infection), two (death)
Almamar et al. 2018 [21]	Robotic	50	205 ± 70	47, 94%	9: Open	0	14	Median 6	Six (surgical site infection), two (respiratory complications), one (cardiac complications), two (others), and three (retained stones)
	Open	30	174 ± 73	28, 93%	0	0	15	Median 12	Seven (surgical site infection), one (respiratory complications), one (cardiac complications), four (others), and two (retained stones)
Alkhamesi et al. 2012 [20]	Robotic	19	220 ± 41.26	16, 84.2%	4: Open	3-post-op ERCP	9	Median 4	Two (surgical site infection), three (respiratory complications), one (death), and three (retained stones)
	Open	18	169 ± 65.81	16, 88.9%	0	1-post-op ERCP	10	Median 11	Four (surgical site infection), one (cardiac complications), one (death), and two (retained stones)
Chan et al. 2011 [19]	Robotic	16	239.3 ± 237.3	-	0	0	3	Mean 7, SD 5.69	Two (bile leaks) and one (respiratory complications)
Ji et al. 2011 [18]	Robotic	5	176 ± 32.1	5, 100%	0	0	1	Mean 5.8, SD 2.5	One (respiratory complications)

Regarding surgical approach, most studies performed choledochotomy, two focused on transcystic approach, and one used both. The majority of studies performed robotic CBD exploration alongside cholecystectomy, favoring a single-stage approach for the definitive management of both gallbladder and common bile duct pathology.

Risk of Bias Assessment

**Figure 2:**
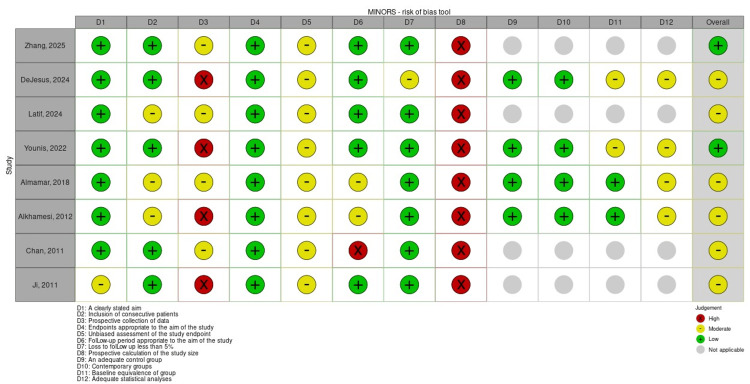
Methodological quality assessment using MINORS criteria for all studies. Individual studies by Zhang et al. 2025 [25], DeJesus et al. 2024 [24], Latif et al. 2024 [23], Younis et al. 2022 [22], Almamar et al. 2018 [21], Alkhamesi et al. 2012 [20], Chan et al. 2011 [19], and Ji et al. 2011 [18]. MINORS: Methodological Index for Non-Randomized Studies.

The methodological quality of the eight included studies was assessed using the MINORS criteria: two studies had an overall low risk of bias, and six had a moderate risk of bias. The results are summarized in Figure 2.

Most studies demonstrated a clearly stated aim and collected outcomes appropriate to the study design. Only one study reported a loss to follow-up exceeding 5% of patients. Given the inherent nature of surgical research, blinding of participants is typically not feasible; therefore, all studies received a score of 1 for inadequately reported blinding.

A potential source of bias was the absence of prospective power calculation for determining study size. While two studies reported prospective data collection, it remained unclear whether a formal protocol was established prior to data collection.

Most studies set a three-month follow-up period, which is appropriate to the aim of the study. However, one study did not report any long-term follow-up, which is crucial for assessing efficacy. In that study, while most patients underwent procedures for suspected CBD stones, it was unclear whether the presence of stones was confirmed at the end of the procedure [19].

For comparator studies, one study showed selection bias, as patients were referred for RACBDE after failed ERCP by surgeons and endoscopists, suggesting the inclusion of more complicated or unwell patients [22]. Another study included subjects who failed initial management pathways and were cleared through the alternate technique, which may have led to duplication in the cohort [24].

*Single-Arm Analysis of Robotic-Assisted Common Bile Duct Exploration* (*RACBDE)*

**Figure 3:**
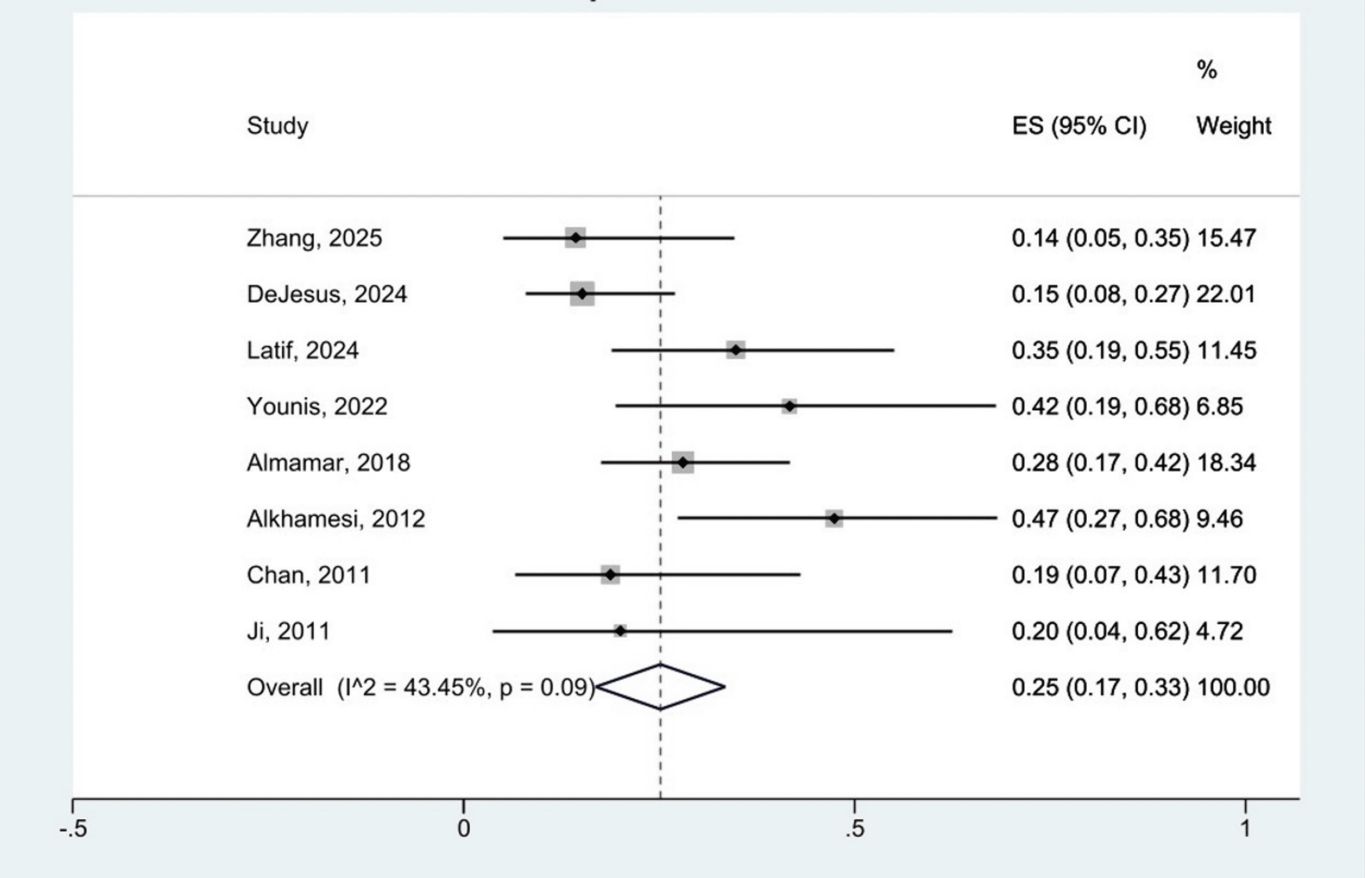
Forest plot showing overall complication of RACBDE for single-arm analysis. Individual studies by Zhang et al. 2025 [25], DeJesus et al. 2024 [24], Latif et al. 2024 [23], Younis et al. 2022 [22], Almamar et al. 2018 [21], Alkhamesi et al. 2012 [20], Chan et al. 2011 [19], and Ji et al. 2011 [18]. RACBDE: robotic-assisted common bile duct exploration.

**Figure 4:**
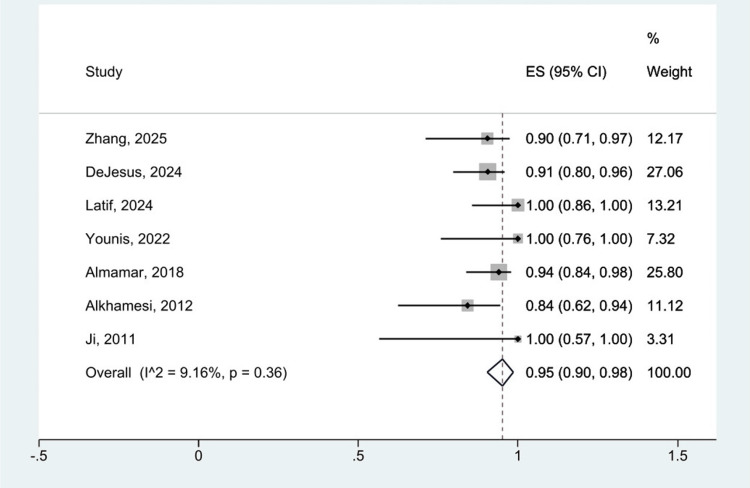
Forest plot showing overall stone clearance rate of RACBDE for single-arm analysis. Individual studies by Zhang et al. 2025 [25], DeJesus et al. 2024 [24], Latif et al. 2024 [23], Younis et al. 2022 [22], Almamar et al. 2018 [21], Alkhamesi et al. 2012 [20], Chan et al. 2011 [19], and Ji et al. 2011 [18]. RACBDE: robotic-assisted common bile duct exploration.

Single-arm analysis was used initially to evaluate efficacy and safety outcomes. Out of eight datasets, the overall pooled complication rate associated with RACBDE was 25% (95% CI: 0.17,033) with moderate heterogeneity (I^2 ^= 43.45%, p = 0.09). This is shown in Figure 3. Equally, the pooled stone clearance rate demonstrated high efficacy, reaching 95% (95% CI: 0.90, 0.98), with low heterogeneity (I^2 ^= 9.16%, p = 0.36). This is displayed in Figure 4. All data analysis was performed using a random effects model and a 95% confidence interval.

Comparative Analysis of Robotic-Assisted Common Bile Duct Exploration (RACBDE)​​​​​​​ Versus Open Surgery

**Figure 5:**
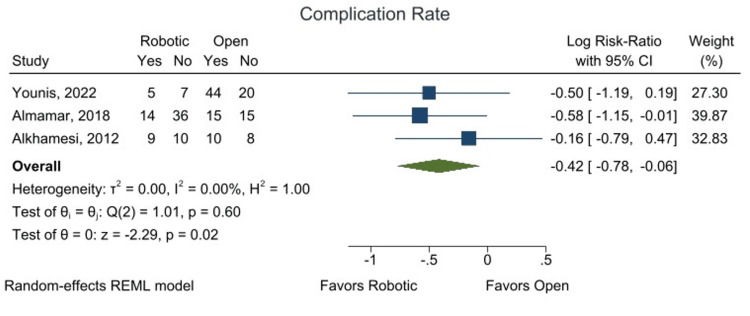
Forest plot showing overall complication rate using pooled log risk-ratio from comparative analysis. Individual studies by Younis et al. 2022 [22], Almamar et al. 2018 [21], and Alkhamesi et al. 2012 [20].

RACBDE was associated with a statistically significant lower risk of complications compared to open surgery. The logarithmic Relative Risk (log RR) was -0.42 (95% CI: -0.78 to -0.06; p < 0.05), as shown in Figure 5. When converted to a relative risk (RR), this indicates that RACBDE confers approximately a 34% lower risk of complications (RR: 0.66; 95% CI: 0.46-0.94) compared to open surgery.

**Figure 6:**
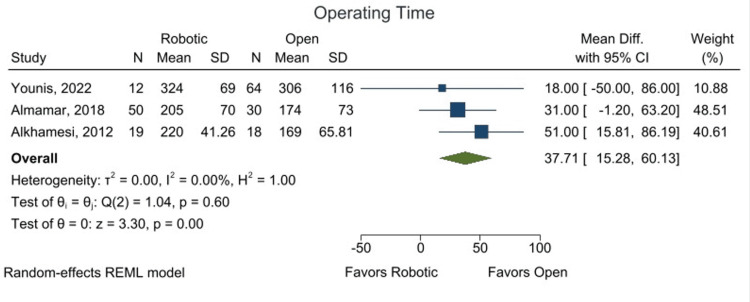
Forest plot showing operating time using mean difference from comparative analysis. Individual studies by Younis et al. 2022 [22], Almamar et al. 2018 [21], and Alkhamesi et al. 2012 [20].

Despite the lower complication rate, open surgery demonstrated a significantly shorter operative time. The mean difference (MD) in operative time was 37.71 minutes (95% CI: 15.28-60.13; p < 0.05), as shown in Figure 6, indicating that open surgery was on average 37 minutes faster than RACBDE.

When comparing stone clearance rate, there was no statistically significant difference between RACBDE and open surgery (log RR: -0.18, 95% CI: -1.31 to 0.96, p = 0.76).

Meta-Regression Analysis

**Figure 7:**
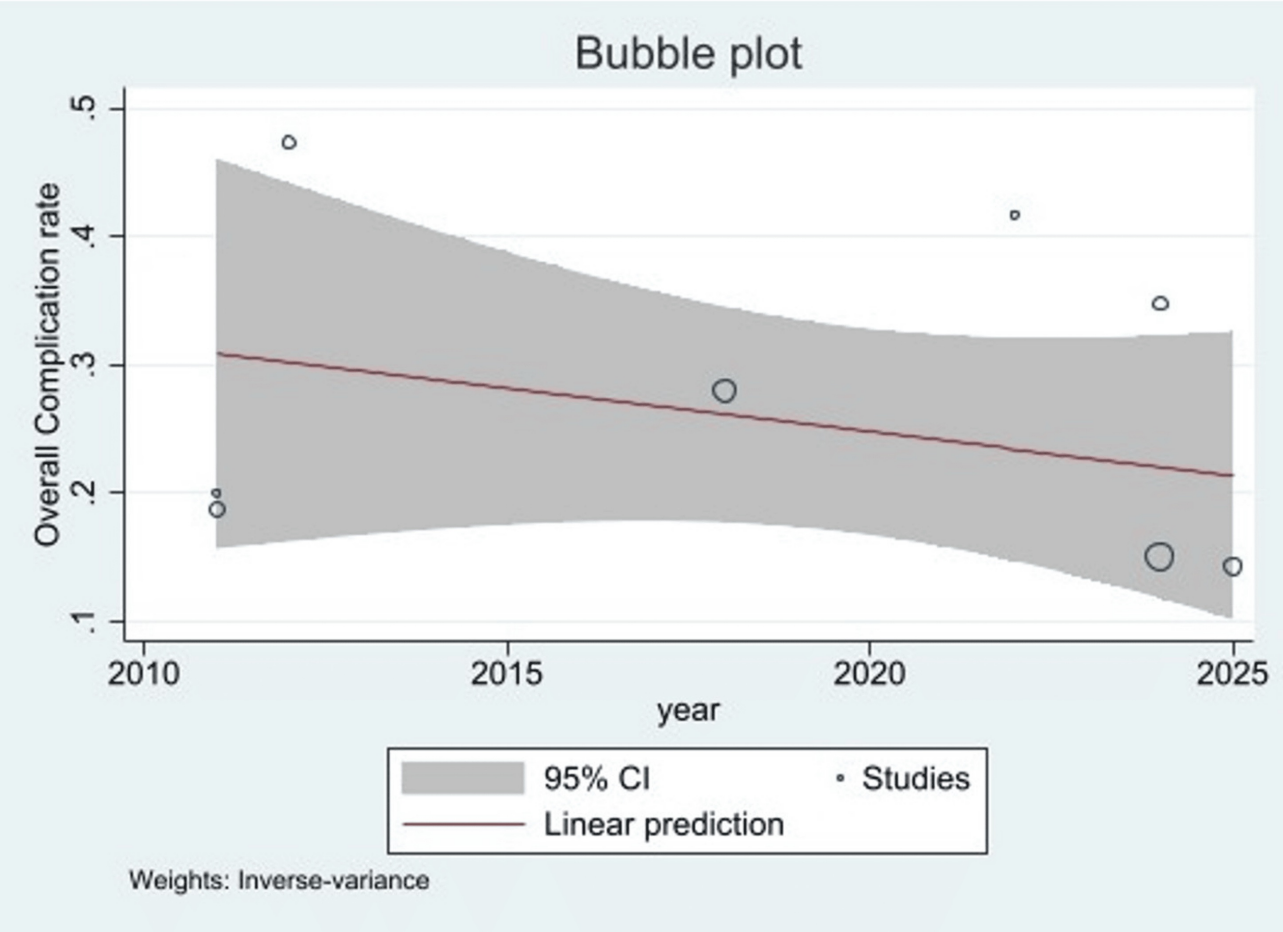
Bubble plot showing meta-regression of overall complication rate with year as covariate.

Univariable meta-regression with publication year as covariate showed a reduction in overall complication rate by 0.7% per year (coefficient: -0.00679, p = 0.37). However, this was not statistically significant (Figure 7).

Multivariable meta-regression on overall complication rate with different variables, including sample size, operation time, and surgical approach, as shown in Table 3, showed no statistically significant results.

**Table 3:** Multivariable Regression with different variables

Variable	Coefficient	P-value
Sample size	+0.0012	0.814
Operation Time	+0.0013	0.475
Surgical approach	+0.1622	0.232

Discussion

Our findings suggest that RACBDE is a highly effective surgical approach with an acceptable complication rate of 25% and a high pooled stone clearance rate of 95%. When compared to other treatment approaches, these results remain promising.

For instance, a large multicenter study involving 1,689 patients reported an overall complication rate of 18.9% for laparoscopic common bile duct exploration (LCBDE) [27]. Similarly, a systematic review on LCBDE found that most studies reported stone clearance rates above 84% [28]. Another meta-analysis comparing one-stage LCBDE with two-stage ERCP followed by cholecystectomy found stone clearance rates of 90% and 84% respectively, with overall complication rates of 45% for one-stage and 40% for two-stage approaches [29].

Additionally, a meta-analysis that looked at percutaneous transhepatic fluoroscopy-guided management (PTFM) revealed overall complication rate of 13.8% and stone clearance rate of 97.1% [30]. These figures highlight that RACBDE achieves a stone clearance rate on par with, or even better than, many alternative procedures, while maintaining a complication rate within an acceptable range.

When compared to open CBD exploration, RACBDE demonstrated significantly lower complication rate while maintaining equal success in stone clearance. Current literature describes a wide range of complication rates from 3 to 41% for open choledochotomy [31]. To our knowledge, this is the first meta-analysis to review efficacy and safety outcomes of RACBDE.

These results are particularly relevant in the context of an increasing shift toward minimally invasive, single-stage procedures for managing both gallbladder and bile duct stones. As demonstrated in our analysis, most included studies performed CBD exploration concurrently with cholecystectomy, showing that this single-stage approach is both practical and commonly used. This strategy has been found to provide higher CBD stone clearance, shorter hospital stays, and lower costs compared to multi-stage procedures [8,32].

While our meta-analysis found that operating time was significantly longer in the robotic group compared to open or laparoscopic approaches, this is largely due to the steep learning curve encountered when adopting robotic platforms. Early studies have compared experienced laparoscopic surgeons, who were still learning how to use the robotic system. This difference in experience likely influenced the outcomes and may have introduced bias.

In a randomized simulation study, novice participants performing suturing tasks using robotic systems achieved substantially faster median times than those using laparoscopy (251 s vs. 611 s), but both groups improved over time, reflecting rapid skill acquisition with robotics [33]. Additionally, one study looking at robotic cholecystectomy suggests that total operating time steadily declines with experience, with docking and console times reducing significantly over the first 30-50 cases [10].

Another key consideration is cost-effectiveness and availability. Robotic surgical platforms require higher capital, ongoing maintenance expenses, and higher disposable instrument costs compared with conventional laparoscopy. In the context of biliary surgery, a Canadian cost analysis reported that the operating room cost of RACBDE was nearly twice that of open surgery; however, this was offset by higher postoperative hospitalization costs in the open group, resulting in a net overall mean hospital cost of approximately $3,000 CAD per RACBDE case [21]. When compared with laparoscopic approaches, current robotic biliary procedures remain more costly and, to date, have not consistently demonstrated superior clinical outcomes to justify the additional expense [34].

Access to robotic surgery is also highly uneven across the globe. Most robotic systems are concentrated in high-income countries, while many low- and middle-income countries face major barriers, including capital investment, maintenance contracts, and the need for specialized training. Despite this, robotic surgery is a rapidly expanding field, with procedural volumes increasing at an estimated 15% annually [35]. Uptake is highest in North America and in certain European centers, while adoption remains limited across South America, Africa, and much of Asia [36].

Despite promising results from this meta-analysis, several limitations warrant consideration when interpreting our findings. Firstly, the number of included studies was small, which comprised of mostly cohort and case-control studies. Among those studies, different comparators were used. As a result, much of the analysis relied on single-arm studies, limiting our ability to draw strong conclusions about the comparative effectiveness of RACBDE. Another limitation is that we only included studies published in English, which might have introduced bias and excluded relevant data from non-English journals. 

Secondly, while cholecystectomy may improve overall clinical outcomes, its inclusion complicates efforts to isolate and evaluate the performance of RACBDE as an independent procedure. Most RACBDE was performed with concurrent cholecystectomy; pooled “overall complications” should be interpreted cautiously; CBDE-specific events (bile leak, stricture, retained stones) offer the fairest estimate of duct-exploration risk.

Furthermore, the optimal treatment surgery for common bile duct stones remains a subject of ongoing debate. Although ERCP with sphincterotomy is widely accepted as first-line due to its minimally invasive nature, it is not always successful, especially in patients with difficult anatomy, impacted stones, or failed cannulation [21,37,38]. In such cases, surgery, including robotic-assisted approaches, is often reserved as a salvage or secondary treatment, rather than a primary modality. This practice inherently biases surgical cohorts towards more complex or refractory cases, potentially undermining the full efficacy and safety potential of RACBDE [31].

Moreover, long-term outcomes, such as stone recurrence, chronic complications, and patient-reported quality of life, were not stated in most studies. While short-term outcomes, such as efficacy and safety, provided valuable insights, they do not fully capture the overall impact of RACBDE. Stone recurrence can occur months or years after the initial procedure, especially in patients with anatomical variations or underlying biliary dyskinesia. Similarly, complications such as stricture formation or recurrent infections may only manifest in the longer term [38,39]. Future studies should report outcomes separately for cholecystectomy and CBDE components and standardize 30/90 day window.

## Conclusions

Our study demonstrates that RACBDE is a highly effective and safe option for the management of choledocholithiasis, with high stone clearance rates and acceptable complication profile when compared to other modalities from existing literature. When compared to open surgery, RACBDE offers significant reduction in postoperative complications. Though it is associated with longer operative times, robotic platforms are expected to improve over time. These findings need to be interpreted with caution due to the limited number of comparative studies, the reliance on single-arm cohorts, and the variability in concurrent procedures, such as cholecystectomy. Further advances in robotic training programs and increased accessibility to robotic surgery will provide new evidence to clarify its efficacy and safety profile.
